# The Utility of Texture Analysis Based on Breast Magnetic Resonance Imaging in Differentiating Phyllodes Tumors From Fibroadenomas

**DOI:** 10.3389/fonc.2019.01021

**Published:** 2019-10-15

**Authors:** Hui Mai, Yifei Mao, Tianfa Dong, Yu Tan, Xiaowei Huang, Songxin Wu, Shuting Huang, Xi Zhong, Yingwei Qiu, Liangping Luo, Kuiming Jiang

**Affiliations:** ^1^Department of Medical Imaging, The First Affiliated Hospital of Jinan University, Guangzhou, China; ^2^Department of Radiology, The Third Affiliated Hospital of Guangzhou Medical University, Guangzhou, China; ^3^Department of Radiology, Shenzhen Maternity and Child Healthcare Hospital, Shenzhen, China; ^4^Department of Radiology, Guangdong Women and Children Hospital, Guangzhou, China; ^5^Paul C. Lauterbur Research Center for Biomedical Imaging, Institute of Biomedical and Health Engineering, Shenzhen Institutes of Advanced Technology, Chinese Academy of Sciences, Shenzhen, China

**Keywords:** texture analysis, breast, magnetic resonance imaging, phyllodes tumor, fibroadenoma

## Abstract

**Background:** The preoperative diagnosis of phyllodes tumors (PTs) of the breast is critical to appropriate surgical treatment. However, reliable differentiation between PT and fibroadenoma (FA) remains difficult in daily clinical practice. The purpose of this study was to investigate the utility of breast MRI texture analysis for differentiating PTs from FAs.

**Materials and Methods:** Forty-two PTs and 42 FAs were enrolled in this retrospective study. Clinical and conventional MRI features (CCMF) and MRI texture analysis were used to distinguish between PT and FA. Texture features were extracted from the axial short TI inversion recovery T2-weighted (T2W-STIR), T1-weighted pre-contrast, and two contrast-enhanced series (first contrast and third contrast). The Mann–Whitney *U* test was used to select statistically significant features of texture analysis and CCMF. Using a linear discriminant analysis, the most discriminative features were determined from statistically significant features. The K-nearest neighbor classifier and ROC curve were applied to evaluate the diagnostic performance.

**Results:** With a higher classification accuracy (89.3%) and an AUC of 0.89, the texture features on T2W-STIR outperformed the texture features on other MRI sequences and CCMF. The AUC of the combination of CCMF with texture features on T2W-STIR was significantly higher than that of CCMF or texture features on T2W-STIR alone (*p* < 0.05). Based on the result of the classification accuracy (95.2%) and AUC (0.95), the diagnostic performance of the combination strategy performed better than texture features on T2W-STIR or CCMF separately.

**Conclusions:** Texture features on T2W-STIR showed better diagnostic performance compared to CCMF for the distinction between PTs and FAs. After further validation of multi-institutional large datasets, MRI-based texture features may become a potential biomarker and be a useful medical decision tool in clinical trials having patients with breast fibroepithelial neoplasms.

## Introduction

Phyllodes tumor (PT) is a rare tumor accounting for 0.3–1.0% of all mammary tumors and comprises 2–3% of all fibroepithelial mammary neoplasms (1, 2). The histological classification is subdivided into benign, borderline, or malignant (3); however, histological type is found to poorly correlate with clinical behavior (4, 5). Incidence of local relapse is high regardless of the histological grading, and distant metastasis may occur in approximately 25% of malignant PTs (6, 7). With similar clinical features and histopathological appearance, PT may mimic a fibroadenoma (FA), which is the most common benign tumor of the breast. Sometimes, even preoperative invasive procedures such as fine-needle aspiration cytology and core needle biopsy may fail to correctly differentiate these two entities, primarily owing to lack of adequate and representative samples (8, 9). Given the different prognosis, a surgical excision is essential with a wide margin of at least 1 cm for all grades of PT to avoid local relapse and subsequent surgery (10, 11); on the other hand, a FA can usually be safely followed-up or managed by a simple enucleation (12). Therefore, accurate preoperative diagnosis is crucial to offer an appropriate clinical strategy, thus avoiding operative complications resulting from inadequate excision or surgical overtreatment.

Clinically, in contrast to FA, PT can grow rapidly to huge bulky ones with a high reported incidence of local relapse (13). In addition, PT was generally thought to develop later in life than FA (6, 14).

According to previous reports, MRI features have been valuable in the differentiation between PTs and FAs. Kamitani et al. (15) described the MRI features of PTs and noticed a pattern of heterogeneous enhancement, internal cystic components, and increased lobulations in PTs. Although certain clinical and MRI features may raise the index of suspicion, it is challenging to make a reliable differentiation between PT and FA. In daily clinical practice, a benign, small-sized borderline or malignant PT can be easily mistaken for a FA, whereas giant FAs may show overlapping MRI features of PTs.

Radiomics has drawn increasing attention in recent years. It is based on a hypothesis that medical imaging information can be converted into quantitative and mineable features via automatically high-throughput extraction of data characterization algorithms that in turn provide valuable diagnostic, prognostic, or predictive assessment (16–18). Several radiomics studies have shown that some quantitative imaging signatures, such as texture features derived from MRI, can provide an opportunity to facilitate better clinical decision-making in oncology at low cost and non-invasively. For example, texture analysis has been used to predict sentinel lymph node metastasis in breast cancer (19), differentiate estrogen receptor-positive breast cancer molecular subtypes (20), and identify healthy breast tissue and breast cancer lesions (21).

Thus, in the present study, we hypothesized that texture features on routine, enhancement, and non-enhanced T1- and T2-weighted MR images, could help to improve the differentiation between PTs and FAs.

## Materials and Methods

### Patients

The retrospective study protocol was approved by our institutional review board. In this study, 53 female patients with histologically confirmed PT between June 2012 and June 2018 were enrolled and 78 female patients with histologically confirmed FA were randomly selected. The inclusion criteria were as follows: (1) female patients were histologically diagnosed with PT or FA by two experienced pathologists based on findings in the specimens obtained at surgical resection, (2) those who underwent breast MRI prior to surgical resection, and (3) those with lesions measuring >1 cm in diameter avoiding the possible unfavorable effects on textural features extracted from image data. The exclusion criteria were as follows: (1) a previous history of breast cancer and radiotherapy, and (2) poor image quality. Finally, 41 female patients with 42 PTs and 37 female patients with 42 FAs were eligible in this study.

### MRI Acquisition

All patients were scanned using a 1.5T dedicated breast MRI system (Aurora Dedicated Breast MRI Systems) with a single channel breast coil. For dynamic imaging, gadolinium-diethylenetriamine pentaacetic acid (Gd-DTPA, Magnevist) was intravenously injected as a bolus of 0.2 ml per kg of body weight at a rate of 2 mL/s followed by a 20-mL normal saline flush. A dynamic series of transverse T1-weighted fat-suppression images were acquired at pre-contrast and post-contrast at 90, 270, 450, and 630 s by using the following imaging parameters: TR = 29 ms, TE = 4.8 ms, slice thickness = 1.1 mm, matrix = 360 × 360 × 128, and FOV = 36 cm. In addition, axial short TI inversion recovery T2-weighted (T2W-STIR) images were performed under the following conditions: TR = 6,680 ms, TE = 68 ms, slice thickness = 3.0 mm, matrix = 320 × 192, FOV = 36 cm. Fat suppression was applied using a short TI-inversion recovery technique.

### Clinical and Conventional MRI Features Assessment

**Figure 1:**
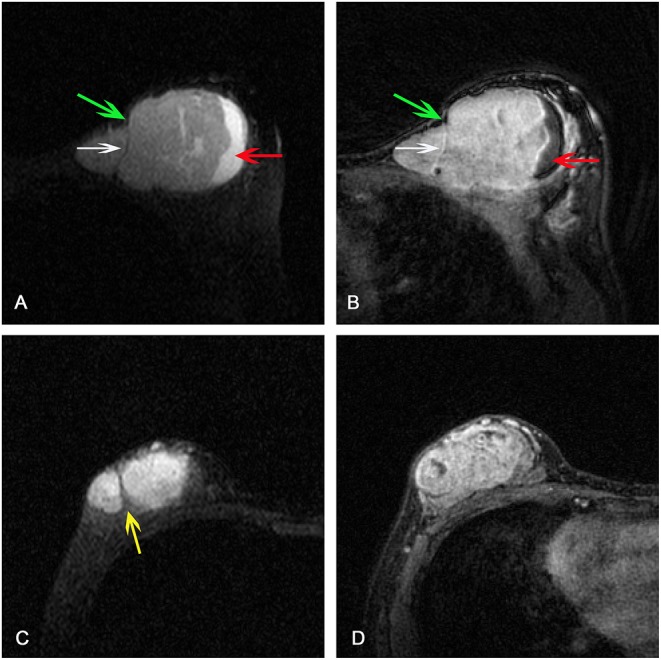
**(A)** Axial short TI inversion recovery T2-weighted (T2W-STIR) and **(B)** third post-contrast image showing a mass with cystic component (red arrow), weak lobulation with obtuse angle (green arrows), septation (white arrow), and heterogeneous enhancement. Strong lobulation with acute angle (yellow arrow) was detected on **(C)** T2W-STIR and heterogeneous enhancement was detected on **(D)** third post-contrast image.

Clinical and conventional MRI features (CCMF) was used to differentiate PTs from FAs. The clinical variables assessed included age, whether the lesions showed rapid enlargement, and whether the lesions were primary or recurrent. The conventional MRI features for each patient were independently reviewed by two radiologists with 12 and 5 years of experience, respectively, blinded to the histopathological diagnoses. For the cases with discrepancies in the CCMF assessment between the two radiologists, these were jointly reviewed by the two radiologists to reach a consensus for further analysis. Interpretation of some conventional MRI features was based on three following characteristics as per the American College of Radiology Breast Imaging Reporting and Data System MR imaging criteria (version 5) (22, 23): the margin of masses (circumscribed vs. non-circumscribed); initial signal intensity enhancement (slow, medium, or fast); and time–intensity curve (TIC) pattern on dynamic contrast-enhanced images (the persistent, plateau, or washout pattern). The presence or absence of a cystic component and internal septation were determined, and the extent of lobulation was divided into strong (with an acute angle) or weak (obtuse angle). In addition, we analyzed the tumor size (the greatest lesion diameter); tumor signal intensity on T2W-STIR (homogeneous vs. heterogeneous); and signal intensity enhancement of third sequence of post-contrast (homogeneous vs. heterogeneous). For the measurements of enhancement features including initial signal intensity enhancement and TIC, the region of interest (ROI) was placed onto the area of the lesion where the enhancement was strongest in the first sequence of the post-contrast image. Examples of these MRI features were shown in Figure 1. For recurrence patients, only clinical and MRI data at the time of recurrence was included and evaluated in this study.

### Texture Analysis

The T2W-STIR, T1-weighted pre-contrast, and two contrast-enhanced series were chosen for texture analysis. Image slices were selected on the basis of presentation of the largest lesion diameter. The ROI, containing the entire visible tumor and excluding equivocal normal breast tissue, was manually drawn for each image. Texture analysis was performed by software package MaZda 4.60 (The Technical University of Lodz, Institute of Electronics) (24, 25).

MaZda allows the quantitative analysis of approximately 300 texture features based on the following algorithms: histogram, absolute gradient, run length matrix, co-occurrence matrix, autoregressive model, and wavelet transform (24, 25), as shown in Table 1. All these texture features were calculated for each ROI. The co-occurrence matrix parameters were calculated in four directions (θ = 0, 45, 90, and 135°) with interpixel distances of *n* = 1, 2, 3, 4, and 5. The gray-level normalization, which is known to minimize the effect of contrast variation and brightness, was carried out using a method that normalizes image intensities within μ ± 3σ (μ, gray-level mean; and σ, gray-level standard deviation).

**Table 1:** Texture features used summary.

**Algorithm**	**Texture features**
Histogram	Mean, variance, skewness, kurtosis, percentiles 1, 10, 50, 90, and 99%
Absolute gradient (GrM)	Mean, variance, skewness, kurtosis, and percentage of pixels with non-zero gradient
Co-occurrence matrix (COM)	Angular second moment, contrast, correlation, sum of squares, inverse difference moment, sum average, sum variance, sum entropy, entropy, difference variance and difference entropy; parameters computed for 4 directions: (a, 0), (0, a), (a, a), (a, –a) and 5 distances: a = 1, 2, 3, 4, 5, between image pixels
Run-length matrix (RLM)	Run-length non-uniformity, gray-level non-uniformity, long-run emphasis, short run emphasis, and fraction of image in runs; parameters computed for horizontal, 45°, vertical, and 135° directions
Autoregressive model (ARM)	Model parameter vector includes 4 parameters; Sigma: standard deviation of the driving noise
Wavelet	Energy of the wavelet coefficients in sub-bands

In MaZda, a combination of feature selection algorithms including mutual information, classification error probability combined with average correlation coefficients, and Fisher coefficient were applied to determine 30 texture parameters with the highest discriminative power for classification on each MRI pulse sequence. These features were then exported for further processing and classification to a statistical program B11 (24).

### Feature Selection and Classification

Statistically significant features were selected among the raw texture features on each MRI sequence and CCMF. A linear discriminant analysis was performed for statistically significant features using MaZda to obtain the most discriminative features (26). Then, the K-nearest neighbor classifier (K = 3) was employed to distinguish between PT and FA based on the most discriminative features using software routines written in MATLAB 7 (Mathworks). For training the classifier, 28 PTs and 28 FAs were used, whereas for testing the classifier, the remaining 14 PTs and 14 FAs were used.

**Figure 2:**
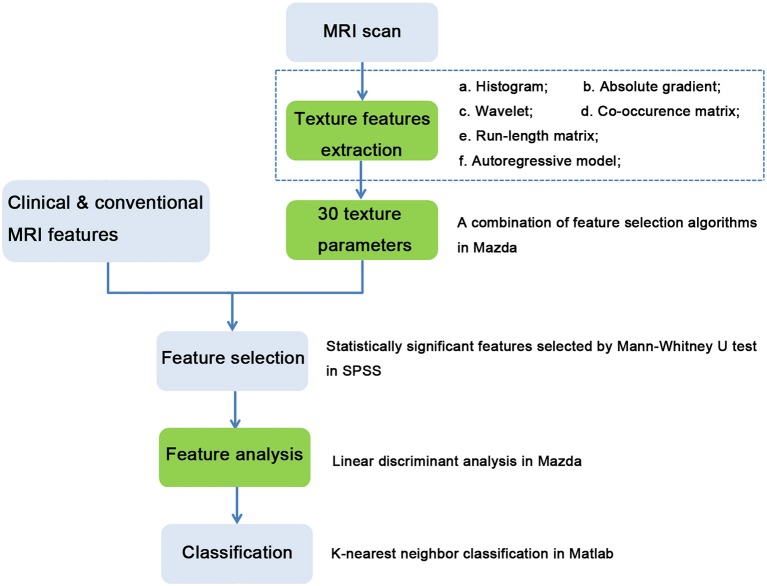
Workflow chart of distinction between phyllodes tumors and fibroadenomas based on clinical and conventional MRI features, and texture features. Processes in green boxes were performed in MaZda.

A workflow chart of the distinction between PT and FA based on texture features and CCMF are illustrated in Figure 2.

### Statistical Analysis

We compared the raw texture parameters on each sequence and CCMF between PTs and FAs using the Mann–Whitney *U* test. For evaluating the diagnostic efficiency of each approach, we employed receiver operating characteristic (ROC) analysis. These analyses were performed using package SPSS 22.0 for Windows. For each ROC curve, comparisons of the area under the curve (AUC) were performed with methods described by Hanley (27). *P* < 0.05 was considered to indicate statistical significance.

## Results

### Clinical and Conventional MRI Features

The clinical characteristics and conventional MRI findings of 42 PTs and 42 FAs are summarized in Table 2. There were 25 benign, 14 borderline, and 3 malignant PTs based on the histological findings. Patients with PTs were significantly older than those with FAs *(p* < 0.001). The mean maximal diameter (standard deviation) was 4.70 ± 3.45 cm for the PT group and 3.48 ± 2.36 cm for the FA group. The PTs tended to be larger than the FAs, although this difference was not statistically significant (*p* > 0.05). The local recurrence rates of PTs and FAs were 26.2 and 0%, respectively. Overall, 61.9% PTs (26/42) showed strong lobulation, whereas only 13 (31.0%) FAs among 42 expressed strong lobulation. The PTs showed strong lobulation pattern more frequently than FAs (*p* = 0.004). Cystic components were seen in 20 (47.6%) PTs but only in 6 (14.3%) FAs (*p* = 0.001). The PTs had a significantly higher frequency of internal septum than the FAs (*p* = 0.009). The FAs tended to be homogeneous more frequently seen on T2W-STIR than the PTs (*p* = 0.001). There were no significant differences between the PTs and FAs in rapid growth, margin, septation enhancement, tumor signal intensity on the third post-contrast images, initial signal intensity enhancement, and TIC curve pattern.

**Table 2:** Clinical and conventional MRI features of phyllodes tumors and fibroadenomas.

	**PT**	**FA**	***P-*value**
**Mean age (SD)**	44.38 ± 6.72	35.07 ± 12.90	<0.001
**Rapid enlargement**			
Absent	30 (71.4%)	33 (78.6%)	0.614
Present	12 (28.6%)	9 (21.4%)	
**Primary/recurrence**			
Primary	31 (73.8%)	42 (100%)	<0.001
Recurrence	11 (26.2%)	0 (0)	
**Diameter**	4.70 ± 3.45	3.48 ± 2.36	0.07
**Margin**			
Circumscribed	32 (76.2%)	34 (81.0%)	0.79
Not circumscribed	10 (23.8%)	8 (19.0%)	
**Strong lobulation**			
Absent	16 (38.1%)	29 (69.0%)	0.004
Present	26 (61.9%)	13 (31.0%)	
**Septation**			
Absent	15 (35.7%)	27 (64.3%)	0.009
Present	27 (64.3%)	15 (35.7%)	
Enhancement	7 (16.7%)	3 (7.1%)	0.312
No enhancement	35 (83.3%)	39 (92.9%)	
**Cystic component**			
Absent	22 (52.4%)	36 (85.7%)	0.001
Present	20 (47.6%)	6 (14.3%)	
**T2W-STIR**			
Homogeneous	22 (52.4%)	36 (85.7%)	0.001
Heterogeneous	20 (47.6%)	6 (14.3%)	
**Initial enhancement**			
Slow	4 (9.5%)	6 (14.3%)	0.636
Medium	15 (35.7%)	17 (40.5%)	
Fast	23 (54.8%)	19 (45.2%)	
**Contrast third**			
Homogeneous	16 (38.1%)	23 (54.8%)	0.126
Heterogeneous	26 (61.9%)	19 (45.2%)	
**TIC pattern**			
Persistent pattern	17 (40.5%)	22 (52.4%)	0.367
Plateau pattern	17 (40.5%)	16 (38.1%)	
Washout pattern	8 (19.0%)	4 (9.5%)	

For clinical and conventional MRI features (CCMF), the classification accuracy of K-nearest neighbor classifier was 76.2%. For ROC analysis, the AUC was 0.76 (95% CI: 0.66, 0.87), and the sensitivity and specificity were both 76.2%.

### Texture Features

PTs and FAs presented a differential textural pattern. Certain texture features extracted using MaZda were significantly different, as shown in Table 3 and Supplementary Information. The number of statistically significant texture features on T2W-STIR was greater than other MRI sequences. For texture features on MRI, the classification accuracies were 89.3, 69.1, 71.4, and 67.9%, for T2W-STIR, T1-weighted pre-contrast, and two contrast-enhanced series (first and third post-contrast sequence), respectively. For ROC analysis, the AUCs were 0.89 (95% CI: 0.82, 0.97); 0.69 (95% CI: 0.58, 0.81); 0.71 (95% CI: 0.60, 0.83); and 0.68 (95% CI: 0.56, 0.80) for T2W-STIR, T1-weighted pre-contrast, and the first and third post-contrast sequences, respectively. The most discriminative features on T2W-STIR had higher classification accuracy (89.3%); AUC (0.89, 95% CI: 0.82, 0.97); sensitivity (88.1%); and specificity (90.5%) than those on other MRI sequences. The result of K-nearest neighbor classifier and ROC analysis are listed in Table 4.

**Table 3:** Statistically significant texture features on axial short TI inversion recovery T2-weighted images.

**Texture feature**	***P***	**Z**
WavEnHH_s-3	<0.001	−3.757
WavEnHH_s-1	<0.001	−4.258
WavEnHL_s-1	0.002	−3.042
GrKurtosis	<0.001	−4.634
GrSkewness	<0.001	−5.573
GrMean	<0.001	−3.569
45dgr_Fraction	<0.001	−4.258
45dgr_ShrtREmp	<0.001	−4.169
45dgr_LngREmph	<0.001	−4.258
S(5,5)SumAverg	0.002	−3.051
S(0,5)SumAverg	0.021	−2.308
S(0,5)InvDfMom	0.003	−2.934
S(4,4)SumAverg	0.003	−2.952
S(4,4)InvDfMom	<0.001	−3.918
S(3,0)Contrast	0.014	−2.460
S(2,2)InvDfMom	<0.001	−3.811
S(2,0)DifVarnc	0.011	−2.541
S(1, −1)DifEntrp	0.004	−2.845
S(1,1)DifEntrp	<0.001	−3.695
S(1,1)InvDfMom	<0.001	−4.053
S(1,1)Correlat	0.002	−3.131
S(1,1)Contrast	0.001	−3.382
S(1,0)DifEntrp	<0.001	−3.543
S(1,0)Correlat	0.001	−3.185
S(1,0)Contrast	0.001	−3.319
Variance	<0.001	−4.348

**Table 4:** Features classification and receiver operating characteristic analysis of phyllodes tumors and fibroadenomas.

	**Classification accuracy**	**AUC (95% CI)**	**Sensitivity**	**Specificity**
T2W-STIR	89.3%	0.89 (0.82, 0.97)	88.1% (37/42)	90.5% (38/42)
Pre-contrast	69.1%	0.69 (0.58, 0.81)	73.8% (31/42)	64.3% (27/42)
First post-contrast	71.4%	0.71 (0.60, 0.83)	71.4% (30/42)	71.4% (30/42)
Third post-contrast	67.9%	0.68 (0.56, 0.80)	66.7% (28/42)	69.0% (29/42)
CCMF	76.2%	0.76 (0.66, 0.87)	76.2% (32/42)	76.2% (32/42)
Combination	95.2%	0.95 (0.90, 1.00)	95.2% (40/42)	95.2% (40/42)

### Combination

For the combination of CCMF with texture features on T2W-STIR, the classification accuracy was 95.2%. The AUC was 0.95 (95% CI: 0.90, 1.00), with a specificity of 95.2% and sensitivity of 95.2%.

### Comparison of Diagnostic Performance

**Figure 3:**
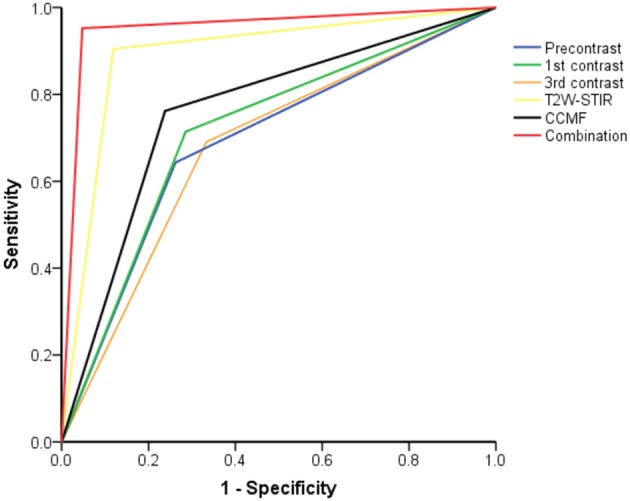
The receiver operating characteristic curves from each approach for differentiation between phyllodes tumors and fibroadenomas.

**Figure 4:**
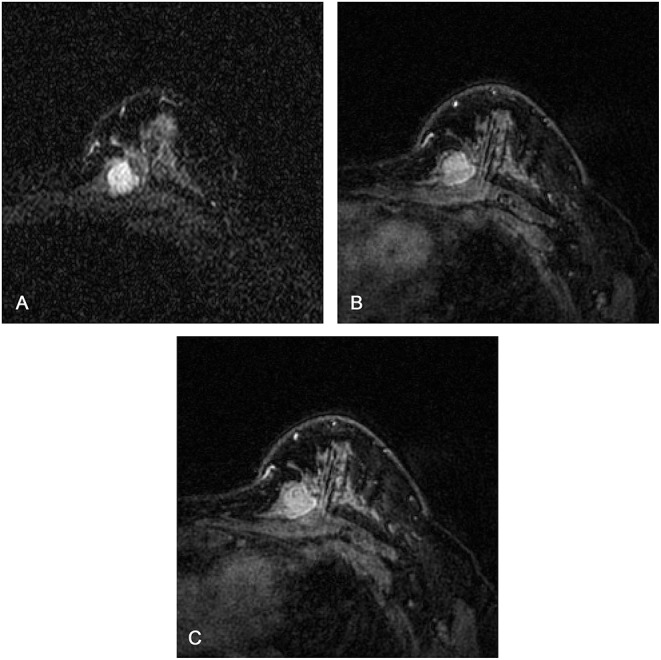
Magnetic resonance images of a 37-year-old female patient with a borderline phyllodes tumor: **(A)** axial short TI inversion recovery T2-weighted (T2W-STIR) **(B)** first post-contrast **(C)** third post-contrast. The texture features on T2W-STIR correctly identified a phyllodes tumor which was falsely interpreted as a fibroadenoma on clinical and conventional MRI features, possibly owing to the weak lobulation, homogeneous signal on T2W-STIR, and absence of cystic component and septation.

**Figure 5:**
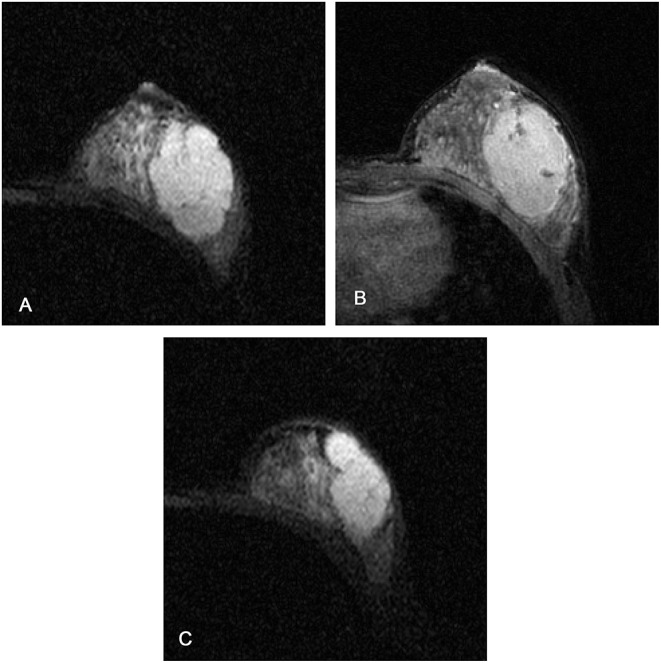
Magnetic resonance images of a 26-year-old female patient with fibroadenomas: **(A,C)** axial short TI inversion recovery T2-weighted (T2W-STIR), **(B)** third post-contrast. The texture features on T2W-STIR correctly identified the fibroadenoma which was falsely interpreted as a phyllodes tumor on clinical and conventional MRI features, possibly due to the cystic component, strong lobulation, and septation.

Figure 3 shows the ROC curves for the K-nearest neighbor classifier when the classifier was trained with most discriminative features of CCMF, texture features on each MRI sequence, and the combination strategy. The texture features on T2W-STIR, with higher classification accuracy (89.3 vs. 76.2%) and AUC (0.89 vs. 0.76), outperformed CCMF. In addition, CCMF was less sensitive than texture features on T2W-STIR (76.2 vs. 88.1%) resulting in a few false negative results (example shown in Figure 4), and exhibited lower specificity (76.2 vs. 90.5%) resulting in a few false positive results (example shown in Figure 5). The AUC of the combination was significantly higher than that of CCMF or texture features on T2W-STIR alone (*p* < 0.05). According to the result of K-nearest neighbor classification and AUC, the diagnostic performance of the combination performed better than texture features on T2W-STIR or CCMF alone.

## Discussion

In the current study, texture analysis based on MRI was applied to evaluate the differential diagnosis between breast PTs and FAs. Texture features on T2W-STIR with higher classification accuracy and AUC performed better than clinical and conventional MRI features (CCMF). Texture features on T2W-STIR were more sensitive than CCMF which exhibited higher specificity. In our study, PT could be mistaken for FA using CCMF but was correctly identified using texture features on T2W-STIR, regardless of whether the lesion was benign or malignant. In addition, we found that the diagnostic performance using the combination of CCMF with texture features based on T2W-STIR was better than CCMF or texture features on T2W-STIR alone. The classification accuracy reached 95.2%, when the most discriminative features of combination strategy were used to train the classifier. By using a combination strategy, the AUC, specificity, and sensitivity were 0.95, 95.2%, and 95.2%, respectively.

Prior studies (15, 28–30) have indicated differences in the clinical and conventional MRI characteristics for differentiating between PTs and FAs, which was also validated in this study. Our study showed that higher age, recurrence, strong lobulation, and internal cystic components were detected significantly more frequently in PTs than in FAs, which were in line with prior studies (15, 28–30). Some groups report that hypointense internal septation was more likely to be presented in PTs than in FAs, but this difference was not statistically significant (15). Even though PTs showed significantly more frequent hypointense internal septations than FAs on MRI in this study, septation enhancement was not statistically significant between PTs and FAs. As reported in some articles (8, 15), PTs were frequently larger than FAs, but there was no significant difference in size between them in our study, likely because the selected tumors were of a relatively large size (>1 cm in diameter). Kamitani et al. (15) found that FAs tended to be homogeneous more frequently than PTs on T1-weighted post-contrast images, but this difference was not significant; there was no significant difference in the TIC curve pattern between the two groups; both of which were consistent with our results. In addition, we found a significantly higher frequency of heterogeneous signals in PTs than in FAs on T2W-STIR, which had been rarely mentioned in previous literatures (15).

Both PTs and FAs are breast fibroepithelial neoplasms. Histologically, they share a dimorphic pattern with both epithelial and stromal components. However, PT can usually be differentiated from FA by its exaggerated intracanalicular growth pattern with increased and heterogeneous stromal cellularity (9). Internal cystic components, septation, and heterogeneous signal on T2W-STIR may be caused by the histopathologically heterogeneous nature of PT, and the stronger lobulation might be related to the rapid growth.

Texture analysis was utilized to evaluate the ability to differentiate PTs from FAs. The number of statistically significant texture features on T2W-STIR was larger than those on T1-weighted pre-contrast and two contrast-enhanced series. Furthermore, the diagnostic performance of these statistically significant texture features on T2W-STIR outperformed that on other MRI sequences, with an AUC of 0.89 and a classification accuracy of 89.3%. The result of texture analysis was in line with that of conventional MRI characteristics that the signal intensity on T2W-STIR was significantly different, but there were no significant differences in features after enhancement between PTs and FAs, such as tumor signal intensity on the third sequence of post-contrast images, initial signal intensity enhancement, and the TIC curve pattern. Previous breast MRI studies mainly focused on dynamic enhancement sequence probably because of its detailed morphological and hemodynamic information; however, dynamic contrast-enhanced MRI was less significant than T2W-STIR to distinguish between PTs and FAs in our study. The echo time of T2W-STIR is relatively long, which offers a higher signal-to-noise ratio, spatial resolution, and soft tissue contrast of breast PTs and FAs. Hence, we hypothesized that texture analysis based on these T2W-STIR images might reveal more subtle alterations in the tumor microenvironment. Textural features extracted from T2W-STIR reflect more differences between PTs and FAs, by capturing the intra-tumoral heterogeneity.

In our study, mean lesion sizes were >3 cm for both PT and FA. With lesion sizes >3 cm, it would be advisable that all these lesions should be resected anyway (8), but they require different surgical procedures. FAs need only enucleation, whereas both benign and malignant PTs require wide local excision with a margin of at least 1 cm (10, 11) because the high recurrence rate in patients with resection margins of <1 cm around the primary tumor (10, 13). A combination of CCMF with texture features on T2W-STIR can provide accurate preoperative diagnosis for these cases with mean sizes >3 cm, which allows appropriate clinical strategy and avoidance of operative complications resulting from inadequate excision or surgical overtreatment.

There were several limitations in our study. First, some of the MRI images were collected after fine needle aspiration of the primary tumor, and thus the hemorrhage or edema caused by the biopsy could have potentially affected feature calculation. Second, we did not explore differences among PTs of all the histologic grading due to the lack of a sufficient number of borderline and malignant PTs. Third, little pathophysiological semantics of the textural features are currently known. Additional work is necessary to understand the underlying biology of these tumors evaluated by texture analysis. Last, as a retrospective study with a small sample size of 84 cases, inherent variations and biases may have influenced the results. Further validation with a larger dataset from different centers and scanners should be strongly considered.

In conclusion, textural features extracted from T2W-STIR showed better diagnostic performance than CCMF. In addition, a combination of CCMF with texture features on T2W-STIR can reflect better diagnostic performance than CCMF or texture features on T2W-STIR alone. Texture analysis provided a novel approach to non-invasively and accurately distinguish PTs from FAs. With ongoing validation, MRI-based texture features may become a potential biomarker and provide a useful medical decision tool in clinical trials in patients with breast fibroepithelial neoplasms.

## Data Availability Statement

All datasets generated for this study are included in the manuscript/Supplementary Files.

## Ethics Statement

The studies involving human participants were reviewed and approved by Institutional Review Board Guangdong Women and Children Hospital. The patients/participants provided their written informed consent to participate in this study.

## Author Contributions

HM, YM, KJ, and LL: conception and design. HM, YM, and KJ: manuscript writing. HM, YM, and TD: provision of study materials or patients. YT, SW, and SH: collection and assembly of data. HM, YM, TD, and YQ: MRI analysis and interpretation. HM, YM, XZ, and XH: statistical analysis. HM, YM, KJ, and LL: final approval of manuscript.

### Conflict of Interest

The authors declare that the research was conducted in the absence of any commercial or financial relationships that could be construed as a potential conflict of interest.

## Abbreviations

PT: Phyllodes tumor
FA: Fibroadenoma
T2W-STIR: Short TI inversion recovery T2-weighted
CCMF: Clinical and conventional MRI features
TIC: time–intensity curve.

## References

[1] Rowell MD Perry RR Hsiu JG Barranco SC. Phyllodes tumors. Am J Surg. (1993) 165:376–9. 10.1016/s0002-9610(05)80849-9 8383473

[2] Liberman L Bonaccio E Hamele-Bena D Abramson AF Cohen MA Dershaw DD. Benign and malignant phyllodes tumors: mammographic and sonographic findings. Radiology. (1996) 198:121–4. 10.1148/radiology.198.1.8539362 8539362

[3] Hoda SA Kaplan RE World Health Organization (WHO) Classification of Breast Tumours, 4th ed. Am J Surg Pathol. (2013) 37:309–10.

[4] Karim RZ Gerega SK Yang YH Spillane A Carmalt H Scolyer RA. Phyllodes tumours of the breast: a clinicopathological analysis of 65 cases from a single institution. Breast. (2009) 18:165–70. 10.1016/j.breast.2009.03.001 19329316

[5] Tan PH Thike AA Tan WJ Thu MM Busmanis I Li H. Predicting clinical behaviour of breast phyllodes tumours: a nomogram based on histological criteria and surgical margins. J Clin Pathol. (2012) 65:69–76. 10.1136/jclinpath-2011-200368 22049216

[6] Reifuss M The treatment of and prognosis of patients with phyllodes tumor of the breast: an analysis of 170 cases. Cancer. (1996) 77:910–6. 8608483 10.1002/(sici)1097-0142(19960301)77:5<910::aid-cncr16>3.0.co;2-6

[7] Bhargav PR Mishra A Agarwal G Agarwal A Verma AK Mishra SK. Phyllodes tumour of the breast: clinicopathological analysis of recurrent vs. non-recurrent cases. Asian J Surg. (2009) 32:224–8. 10.1016/S1015-9584(09)60398-5 19892625

[8] Foxcroft LM Evans EB Porter AJ. Difficulties in the pre-operative diagnosis of phyllodes tumours of the breast: a study of 84 cases. Breast. (2007) 16:27–37. 10.1016/j.breast.2006.05.004 16876413

[9] Jacklin RK Ridgway PF Ziprin P Healy V Hadjiminas D Darzi A. Optimising preoperative diagnosis in phyllodes tumour of the breast. J Clin Pathol. (2006) 59:454–9. 10.1136/jcp.2005.025866 16461806 PMC1860299

[10] Mangi AA Smith BL Gadd MA Tanabe KK Ott MJ Souba WW. Surgical management of phyllodes tumors. Arch Surg. (1999) 134:492–3. 10.1001/archsurg.134.5.487 10323420

[11] Chaney AW Pollack A Mcneese MD Zagars GK Pisters PW Pollock RE. Primary treatment of cystosarcoma phyllodes of the breast. Cancer. (2000) 89:1502–11. 10.1002/1097-0142(20001001)89:7<1502::aid-cncr13>3.0.co;2-p 11013364

[12] Yabuuchi H Soeda H Matsuo Y Okafuji T Eguchi T Sakai S. Phyllodes tumor of the breast: correlation between MR findings and histologic grade. Radiology. (2006) 241:702. 10.1148/radiol.2413051470 17032912

[13] Chen WH Cheng SP Tzen CY Yang TL Jeng KS Liu CL. Surgical treatment of phyllodes tumors of the breast: retrospective review of 172 cases. J Surg Oncol. (2005) 91:185–94. 10.1002/jso.20334 16118768

[14] Cohn-Cedermark G Rutqvist LE Rosendahl I Silfversward C. Prognostic factors in cystosarcoma phyllodes. A clinicopathologic study of 77 patients. Cancer. (1991) 68:2017. 10.1002/1097-0142(19911101)68:9<2017::AID-CNCR2820680929>3.0.CO;2-V 1655234

[15] Kamitani T Matsuo Y Yabuuchi H Fujita N Nagao M Kawanami S. Differentiation between benign phyllodes tumors and fibroadenomas of the breast on MR imaging. Eur J Radiol. (2014) 83:1344–9. 10.1016/j.ejrad.2014.04.031 24856515

[16] Lambin P Leijenaar RT Deist TM Peerlings J de Jong EEC van Timmeren J. Radiomics: the bridge between medical imaging and personalized medicine. Nat Rev Clin Oncol. (2017) 14:749. 10.1038/nrclinonc.2017.141 28975929

[17] Gillies RJ Kinahan PE Hricak H. Radiomics: images are more than pictures, they are data. Radiology. (2016) 278:563–77. 10.1148/radiol.2015151169 26579733 PMC4734157

[18] Kumar V Gu Y Basu S Berglund A Eschrich SA Schabath MB. Radiomics: the process and the challenges. Magn Resonance Imag. (2012) 30:1234–48. 10.1016/j.mri.2012.06.010 22898692 PMC3563280

[19] Dong Y Feng Q Yang W Lu Z Deng C Zhang L. Preoperative prediction of sentinel lymph node metastasis in breast cancer based on radiomics of T2-weighted fat-suppression and diffusion-weighted MRI. Eur Radiol. (2018) 28:582–91. 10.1007/s00330-017-5005-7 28828635

[20] Holli-Helenius K Salminen A Rinta-Kiikka I Koskivuo I Brück N Boström P. MRI texture analysis in differentiating luminal A and luminal B breast cancer molecular subtypes - a feasibility study. BMC Med Imag. (2017) 17:69. 10.1186/s12880-017-0239-z 29284425 PMC5747252

[21] Holli K Lääperi AL Harrison L Luukkaala T Toivonen T Ryymin P. Characterization of breast cancer types by texture analysis of magnetic resonance images. Acad Radiol. (2010) 17:135–41. 10.1016/j.acra.2009.08.012 19945302

[22] Radiology ACo ACR BI-RADS-Magnetic Resonance Imaging. ACR Breast Imaging and Data System, Breast Imaging Atlas (2013).

[23] Rao AA Feneis J Lalonde C Ojeda-Fournier H. A Pictorial Review of Changes in the BI-RADS Fifth Edition. Radiographics. (2016) 36:623–39. 10.1148/rg.2016150178 27082663

[24] Szczypinski PM Strzelecki M Materka A Klepaczko A. MaZda—a software package for image texture analysis. Computer Methods Programs Biomed. (2009) 94:66–76. 10.1016/j.cmpb.2008.08.005 18922598

[25] Castellano G Bonilha L Li LM Cendes F. Texture analysis of medical images. Clin Radiol. (2004) 59:1061–9. 10.1016/j.crad.2004.07.008 15556588

[26] Huang X Zhang Y Qian M Meng L Xiao Y Niu L. Classification of carotid plaque echogenicity by combining texture features and morphologic characteristics. J Ultrasound Med. (2016) 35:2253. 10.7863/ultra.15.09002 27582533

[27] Hanley JA Mcneil BJ. A method of comparing the areas under receiver operating characteristic curves derived from the same cases. Radiology. (1983) 148:839–43. 10.1148/radiology.148.3.6878708 6878708

[28] Plaza MJ Swintelski C Yaziji H Torres-Salichs M Esserman LE. Phyllodes tumor: review of key imaging characteristics. Breast Dis. (2015) 35:79–86. 10.3233/BD-150399 25792027

[29] Brinck U Fischer U Korabiowska M Jutrowski M Schauer A Grabbe E. The variability of fibroadenoma in contrast-enhanced dynamic MR mammography. Am J Roentgenol. (1997) 168:1331–4. 10.2214/ajr.168.5.9129437 9129437

[30] Susanne W Herzog AB Fischer DR Marx C Raabe G Schneider A Differentiation of phyllodes breast tumors from fibroadenomas on MRI. Am J Roentgenol. (2005) 185:1317–21. 10.2214/AJR.04.1620 16247156

