# Supplementary material for: The Utility of Texture Analysis Based on Breast Magnetic Resonance Imaging in Differentiating Phyllodes Tumors From Fibroadenomas
Source: Front Oncol. 2019 Oct 15;9:1021. doi: 10.3389/fonc.2019.01021 (PMC6803552; doi:10.3389/fonc.2019.01021)
Supplement: Supplementary file 1 [file Table_1.DOCX]

Statistically significant texture features on T1-weighted pre-contrast images.

| **Texture feature** | ***P*** | **Z** |
| --- | --- | --- |
| WavEnLH_s-3 | 0.02 | -2.326 |
| GrNonZeros | ˂ 0.001 | -3.637 |
| Vertl_LngREmph | 0.002 | -3.131 |
| Horzl_Fraction | 0.004 | -2.845 |
| Horzl_LngREmph | 0.003 | -2.943 |
| S(0,4)DifVarnc | 0.016 | -2.415 |
| S(0,4)SumVarnc | 0.033 | -2.138 |
| S(0,4)InvDfMom | 0.003 | -2.934 |
| S(0,4)Correlat | 0.004 | -2.863 |
| S(0,4)Contrast | 0.003 | -2.934 |
| S(3,-3)Correlat | 0.02 | -2.326 |
| S(3,3)InvDfMom | 0.003 | -2.997 |
| S(0,3)DifVarnc | 0..01 | -2.585 |
| S(0,3)SumVarnc | 0.021 | -2.308 |
| S(0,3)Correlat | 0.004 | -2.899 |
| S(0,3)Contrast | 0.003 | -2.952 |
| S(2,2)InvDfMom | 0.003 | -3.006 |
| S(0,2)InvDfMom | 0.004 | -2.890 |
| S(0,2)Correlat | 0.002 | -3.051 |

Statistically significant texture features on first post-contrast images.

| **Texture feature** | ***P*** | **Z** |
| --- | --- | --- |
| WavEnLH_s-3 | 0.008 | -2.657 |
| S(5,0)Entropy | 0.014 | -2.451 |
| S(5,0)Entropy | 0.014 | -2.46 |
| S(4,4)Entropy | 0.013 | -2.487 |
| S(4,0)Entropy | 0.01 | -2.576 |
| S(3,-3)Entropy | 0.011 | -2.559 |
| S(3,3)Entropy | 0.011 | -2.55 |
| S(3,0)Entropy | 0.012 | -2.514 |
| S(2,-2)Entropy | 0.011 | -2.55 |
| S(2,-2)AngScMom | 0.013 | -2.487 |
| S(2,2)Entropy | 0.014 | -2.46 |
| S(2,0)Entropy | 0.011 | -2.55 |
| S(1,-1)Entropy | 0.013 | -2.487 |
| S(1,0)Entropy | 0.012 | -2.514 |
| Perc.01% | 0.002 | -3.145 |

Statistically significant texture features on third post-contrast images.

| **Texture feature** | ***P*** | **Z** |
| --- | --- | --- |
| WavEnHL_s-2 | 0.046 | -1.995 |
| WavEnLH_s-2 | 0.011 | -2.559 |
| WavEnHH_s-1 | 0.006 | -2.729 |
| GrNonZeros | 0.002 | -3.095 |
| GrSkewness | 0.001 | -3.319 |
| Vertl_LngREmph | 0.008 | -2.639 |
| S(5,5)SumAverg | 0.027 | -2.21 |
| S(5,5)InvDfMom | 0.006 | -2.737 |
| S(4,4)InvDfMom | 0.018 | -2.362 |
| S(3,3)InvDfMom | 0.008 | -2.648 |
| S(0,3)InvDfMom | 0.004 | -2.872 |
| S(2,2)InvDfMom | 0.001 | -3.435 |
| S(0,2)InvDfMom | 0.006 | -2.746 |
| S(1,1)InvDfMom | 0.003 | -2.979 |
| S(0,1)InvDfMom | 0.007 | -2.693 |
| S(1,0)InvDfMom | 0.026 | -2.228 |
| Perc.01% | 0.003 | -2.93 |
| Skewness | 0.025 | -2.245 |
